# Supplementary material for: Influence of Synthesis Conditions on the Properties of Zinc Oxide Obtained in the Presence of Nonionic Structure-Forming Compounds
Source: Nanomaterials (Basel). 2023 Sep 11;13(18):2537. doi: 10.3390/nano13182537 (PMC10536475; doi:10.3390/nano13182537)
Supplement: Supplementary file 1 [file nanomaterials-13-02537-s001.zip › nanomaterials-2568018-supplementary.pdf]

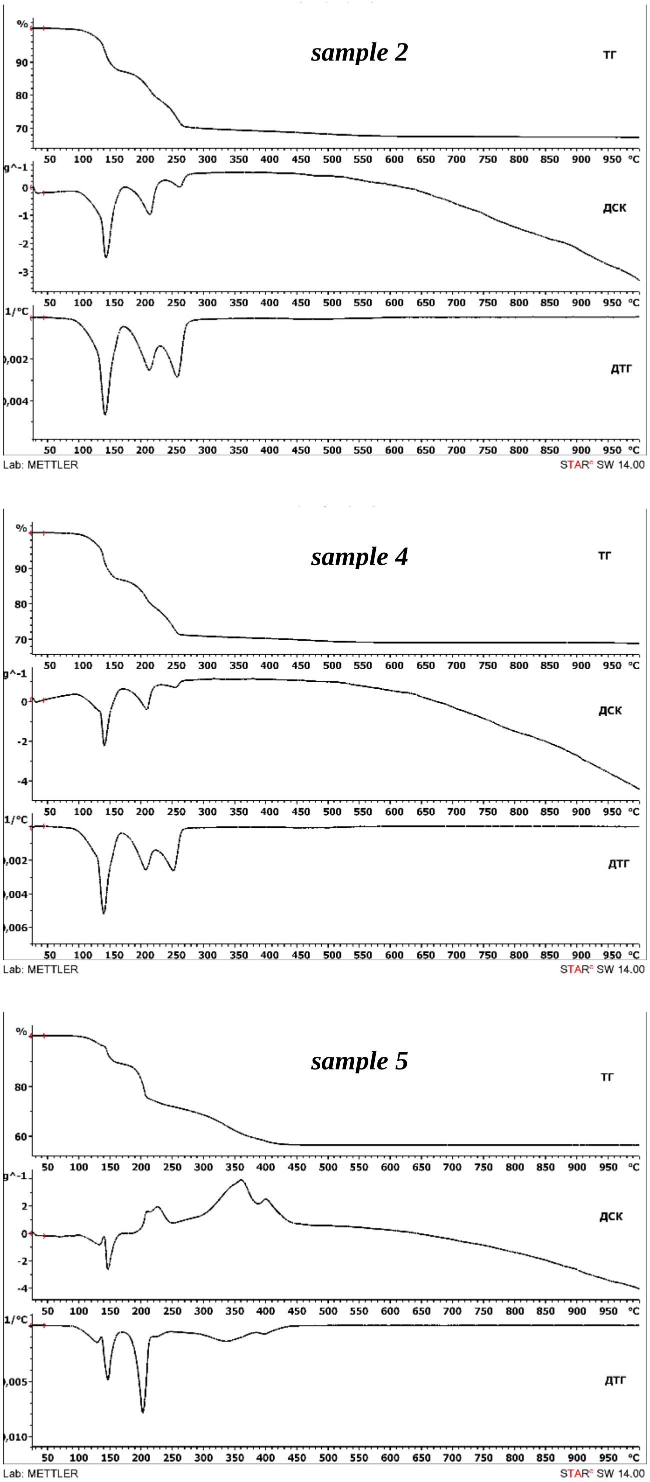

Figure S1 – Thermal analysis data of synthesis intermediate $Zn_5(NO_3)_2(OH)_8{\cdot}2H_2O$ in samples 2, 4, 6

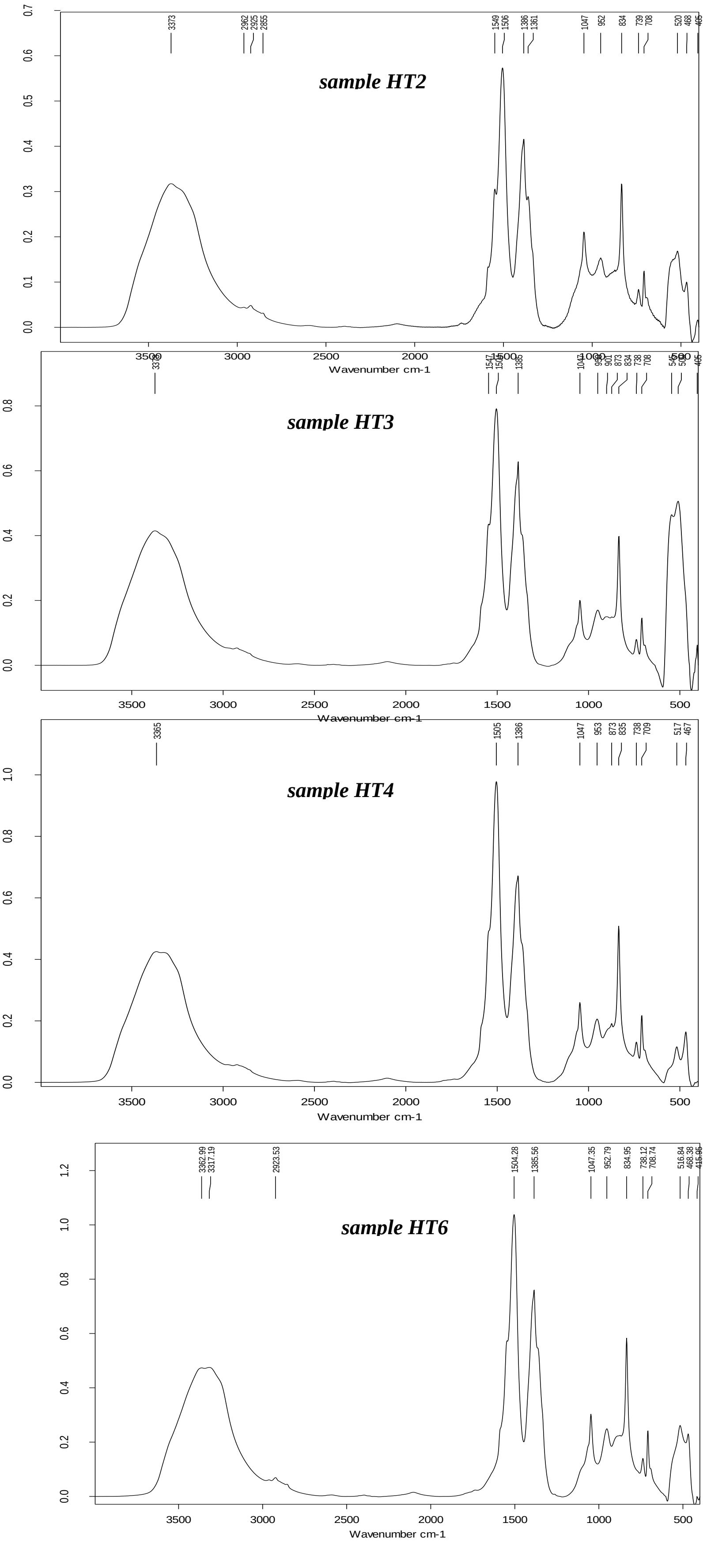

Figure S2 – IR spectra of synthesized zinc oxide samples in the presence of various surfactants

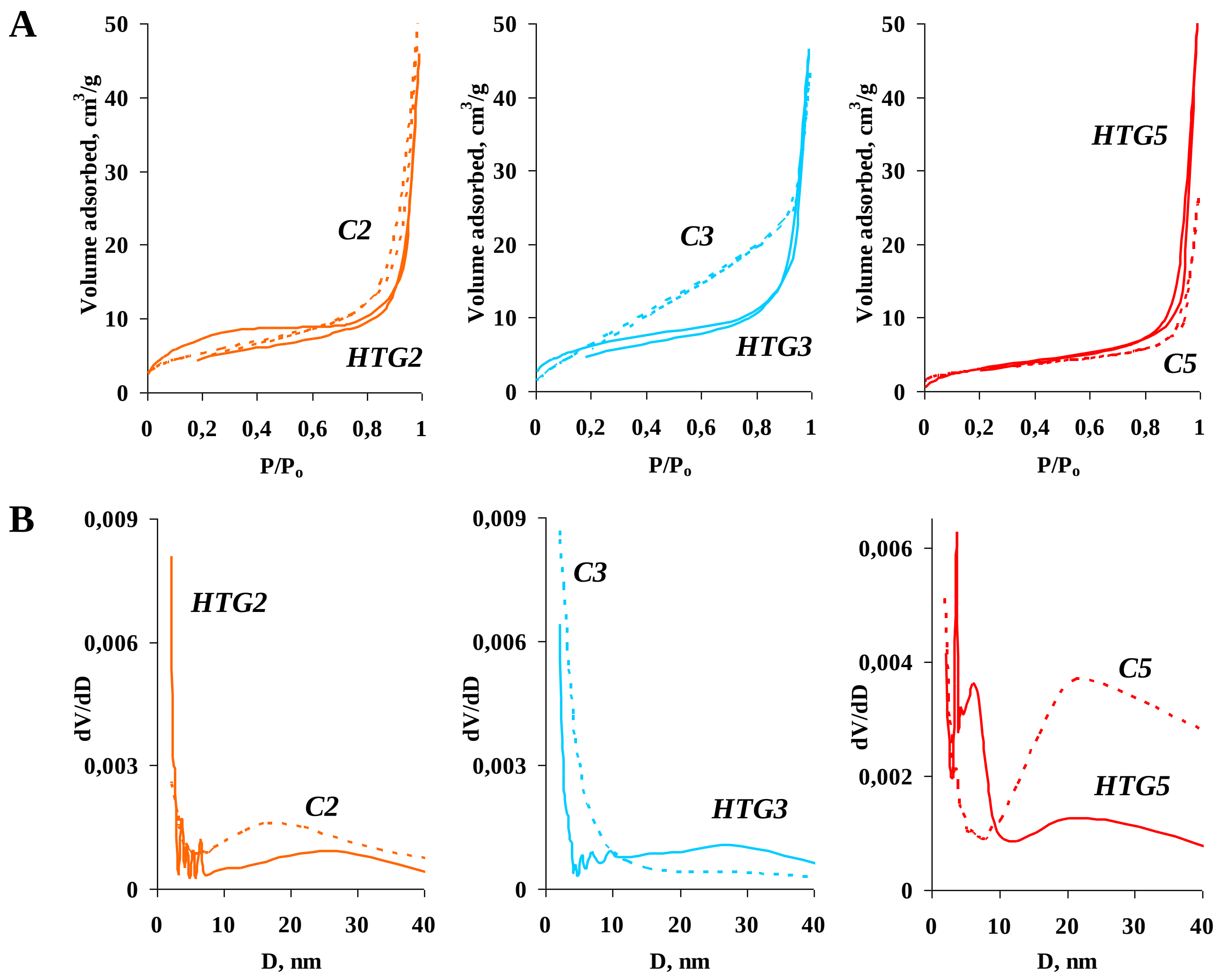

Figure S3 - Sorption isotherms (A) and pore size distribution curves (B) for synthesized samples
